# The history of botulinum toxin in Brazil

**DOI:** 10.5935/0004-2749.20220068

**Published:** 2022

**Authors:** Carlos R. Souza-Dias

**Affiliations:** 1 Faculdade de Ciências Médicas, Irmandade da Santa Casa de Misericórdia de São Paulo, São Paulo, SP, Brazil; 2 Instituto Strabos, São Paulo, SP, Brazil

Botulinum toxin is a neurotoxin produced by the bacteria *Clostridium botulinum* and has always been considered a lethal poison that is acquired via the consumption of contaminated food, such as meat, fish, or pickled vegetables. There are seven different serotypes of botulinum toxin, labeled A, B1, C, D, E, F, and G. This investigation focuses on type A botulinum toxin, as most strains of this type retain their toxigenicity well, yield highly potent culture fluids (≥1 million lethal doses per milliliter of culture fluid), and can easily crystallize in stable form.

The German poet and physician Justinus Andreas Christian Kerner (1786-1862) attended patients with paralysis after ingestion of chorizo. He perceived that the chorizo contained a poison that he termed the “poison of chorizo.” He suggested that that toxin could be used therapeutically in certain muscles. At the beginning of the 20th century, the microorganism responsible for the paralysis was identified as a bacterium, which was named *Clostridium botulinum* (probably based on the Greek word *klostér* [kloster], which means fuse, and the Latin term *botulus*, which means chorizo). At the end of the 1960s, Alan B. Scott, an ophthalmologist and senior investigator of the Smith-Kettlewell Eye Research Institute of San Francisco, California, began to study the therapeutic use of botulinum toxin as part of an investigation for a substance with a lasting but reversible paretic effect for the treatment of infantile strabismus^(1)^. He began his studies in monkeys and then in other animals. He tried some substances, such as alcohol, anesthetics of prolonged action, and snake neurotoxin, until he found type A botulinum, which he named *Oculinum.* His clinical studies with that substance began in 1977 (Figure 1).

**Figure 1:**
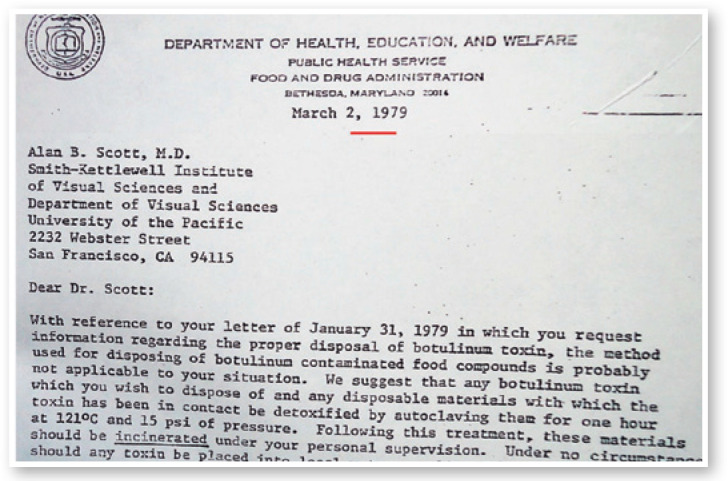
Letter (March 2, 1979) from the Department of Health, Education and Welfare, United States, to Alan B. Scott, about what to do with the contained by botulinum toxin.

In 1988^(2)^, Scott started applying Oculinum to human extraocular muscles for the treatment of strabismus and nystagmus, in addition to the human lid muscle for retraction and blepharospasms. In 1988, the laboratory Allergan bought the rights of the product and later changed its name to *Botox* (Figure 2).

**Figure 2:**
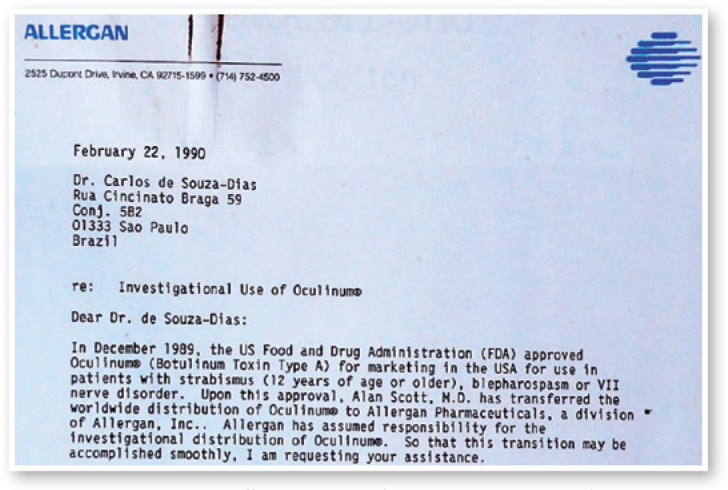
Letter from Allergan to Carlos de Souza-Dias asking for the safety and efficacy data I had collected thus far during my Oculinum treatments.

In view of the need for a profound investigation of the toxin before use in human patients, by exigency of the Food and Drug Administration, Scott named investigators in some countries to increase the statistics of treated patients. In 1981, I was honored with this indication (Figure 3).

**Figure 3:**
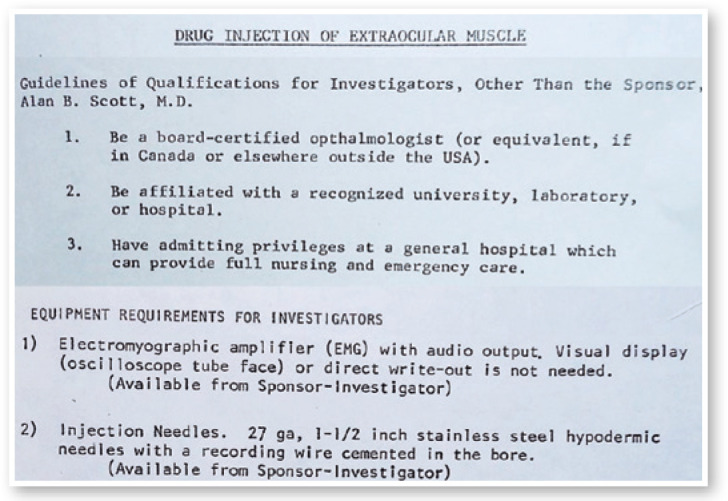
Letter from Alan B. Scott with guidelines of qualifications for investigators, other than the sponsor, and a list of equipment requirements for investigators.

At the beginning, Oculinum was provided to me in San Francisco. I stored it in a Styrofoam box, because it must be kept frozen. The first shipment of the vials was accompanied by an electromyograph and special needles for the injections. Later on, I started receiving the toxin by mail (Figure 4). Each vial contained 250 units to be dissolved in saline solution, according to the desired degree of solution.

**Figure 4:**
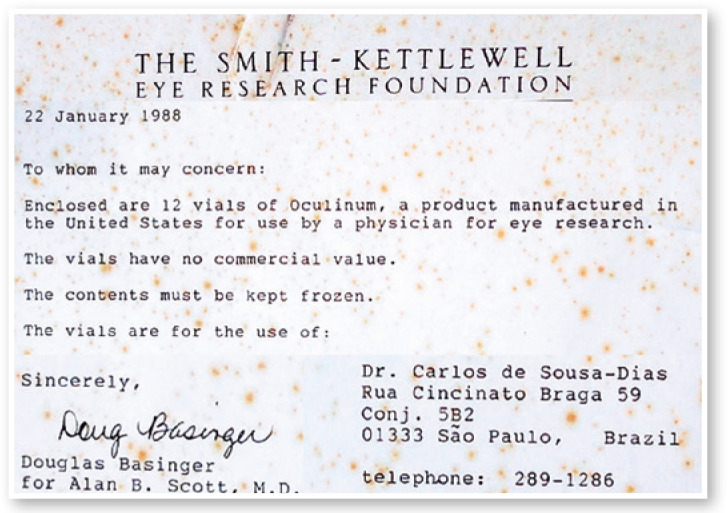
Letter from the Smith-Kettlewell Eye Research Foundation to Carlos de Souza Dias, dated January 22, 1988, together with 12 vials of Oculinum.

For me, at the beginning, it was an investigation, as I had no experience, and the pertinent bibliography was very scarce. I began injecting the toxin in the horizontal recti muscles of patients with esotropia or exotropia in my clinic and in the Santa Casa Hospital of São Paulo, where I was the head of the Ophthalmology Department. I always conducted electro-oculography to observe the degree of the force reduction of the injected muscle (Figure 5).

**Figure 5:**
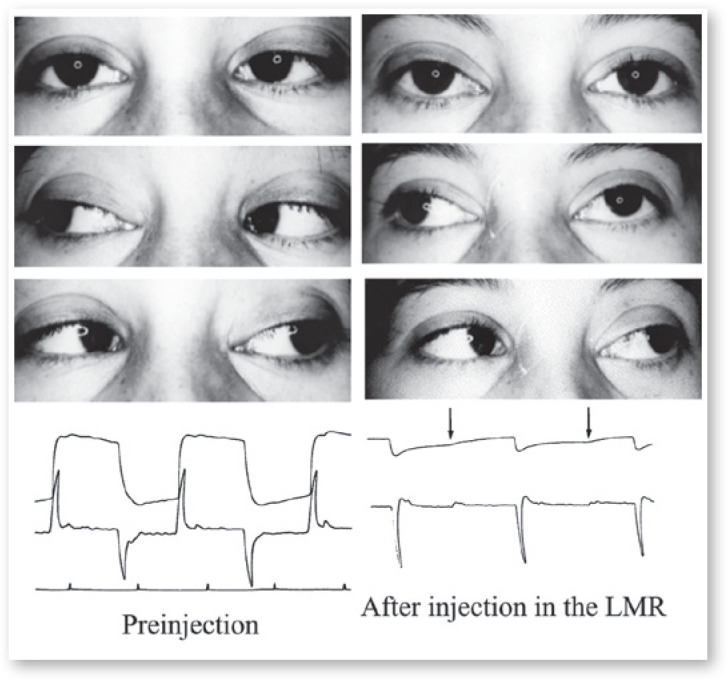
Above, photography of a boy before and after injection of Botulinum in his left medial rectus; it is clear it is muscle paralysis. Lower panels: Electro-oculography before and after Oculinum injection. The images show the reduction in the amplitude and velocity of adduction of the left eye.

I diluted each vial in 2 ml of saline solution and injected between 0.1 and 0.25 ml each time, which corresponds to 1 and 2.5 U (Figures 6 and 7). Here, I present the details of 6 of my first 44 injected patients, which thus represent the first cases in Brazil.

**Figure 6:**
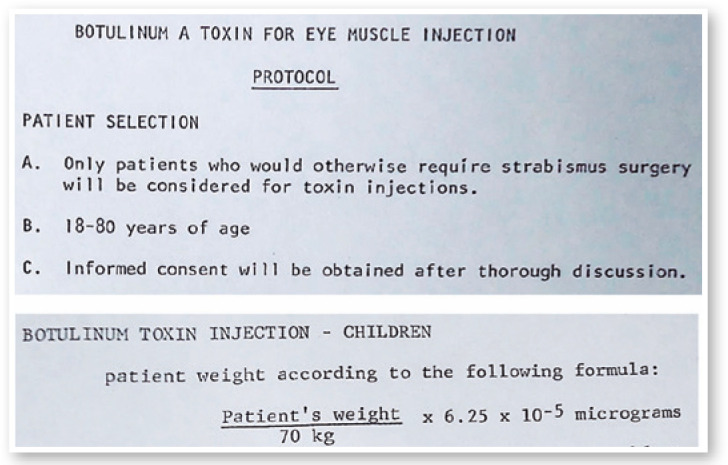
Protocol for Botulinum toxin injection in the eye muscles of patients.

**Figure 7:**
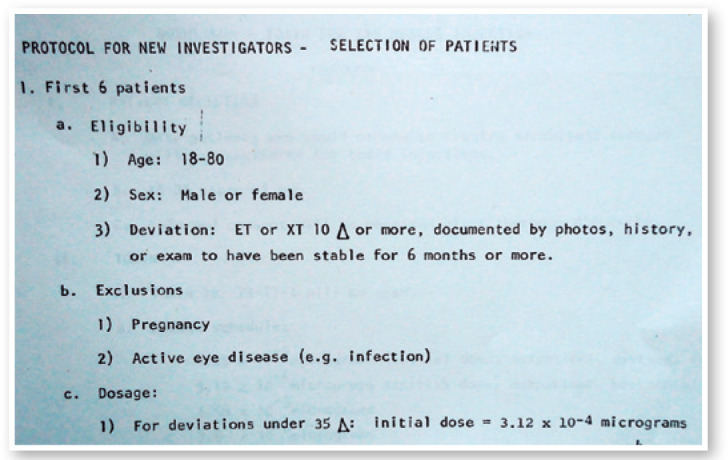
Protocol for new investigators: selection of patients.

## CASE 1

November 15, 1981: A.P., female, 18 years old. The patient had undergone four surgeries for strabismus treatment.

Visual acuity: Both eyes (OU) 20/20.

- Ocular motility:XT 45^D^ RHT 7^D^.
- XT 35^D^ RHT7^D^.
- XT 32^D^ RHT7^D^; MR OU - 2.

November 18, 1981: The patient underwent surgery in OU for LR recession (RLR was 11 mm and RLR was 9 mm from the limbus).

March 26, 1982: XT 30^D^ RHT 6^D^.

March 26, 1982: 0.1 ml of botulinum was injected in the RLR.

March 30, 1982: XT 10^D^ RHT 6^D^; slight blepharoptosis May 19, 1982: XT 13^D^ RHT 8^D^.

October 4, 1982: Oculinum was reinjected in the LLR. October 4, 1982: XT4^D^ RHT 8^D^; esthetically well.

February 4, 1984: XT 17^D^ RHT 12^D^ without anysotropia.

## CASE 2

October 24, 1981: A.F.D., female, 16 years old. retinal detachment of the right eye at 6 years of age.

Visual acuity: Right eye (OD) null and left eye (OS) 0.4 with -16 sph.

Ocular motility: XT 32^D^ RHT 12^D^ without anysotropia.

October 24, 1981: 0.1 ml Oculinum was injected in the RLR.

November 4, 1981: XT 15^D^ RHT 12^D^.

December 12, 1981: XT 20^D^ RHT 12^D^. March 3, 1982: Surgery: RLR recession 6 mm and RMR resection 5 mm with inferior transposition 5 mm.

September 15, 1982: Ortotropia.

## CASE 3

October 24, 1981: S.A.S., female, 20 year old, poor sight in left eye.

Visual acuity: OD 0.9 and OS anisometropic ambliopia with eccentric fixation. Ocular motility:

- ET 45^D^
- ET 40^D^
- ET 50^D^

October 24, 1981: Oculinum was injected in the LMR; eccentric fixation.

**Figure:**
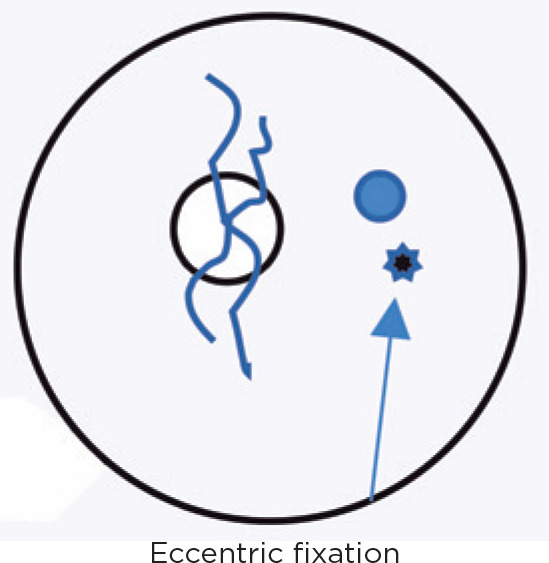

November 11, 1981: No effect; Oculinum was reinjected.

November 27, 1981: XT 15^D^ RLR -4.

Lost for examination.

**Figure 8:**
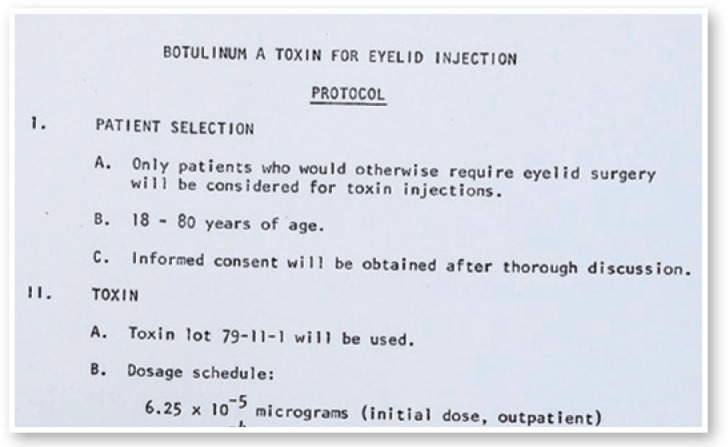
Protocol for eyelid injection.

**Figure 9:**
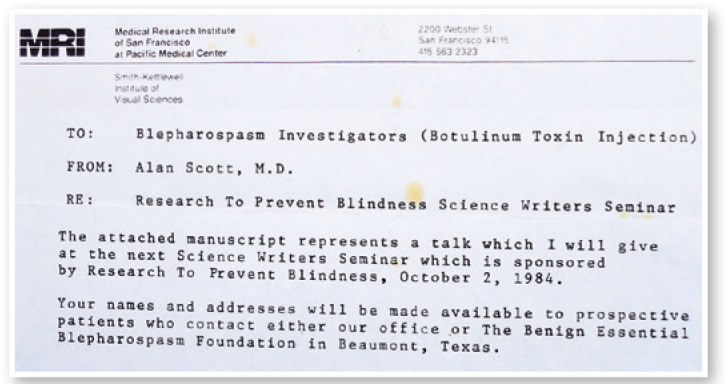
A letter from Alan Scott to the blepharospasm Investigators (Botulinum Toxin Injection) telling me that my name and address would be available to prospective patients after the talk he would give at the Science Writers Seminar, which was sponsored by Research to Prevent Blindness in October 1984.

Among my 44 cases, 14 experienced blepharoptosis. In the beginning, I used the toxin only for correcting correction strabismus, but later on, I started to use it for the treatment of blepharospasm and facial distony^(3)^, after I heard Scott’s presentation about it at the meeting of the International Strabismological Association held in Rome in 1986 (Figures 8 and 9).

## CASE 4

March 7, 1988: S.D.M., 57 years old, male. Four years prior, after the death of his sister-in-law, his left inferior lid started to shake, but he soon began to experience half-face contractions.

**Figure 10:**
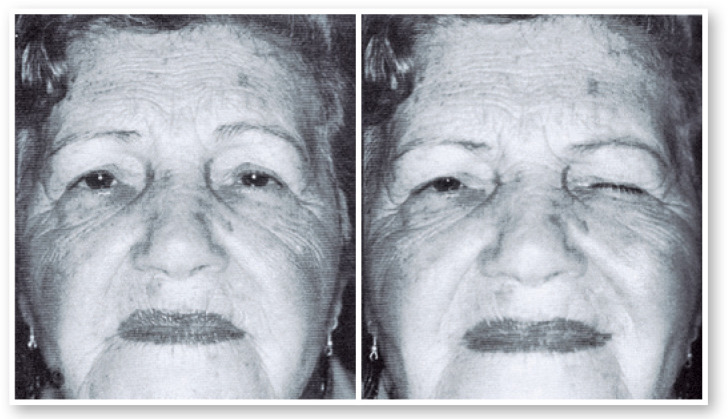
Patient with left eyelid spasm before and after Botox injection.

Diagnosis: Left hemiorofacial spasms (Figure 10).

**Figure 11:**
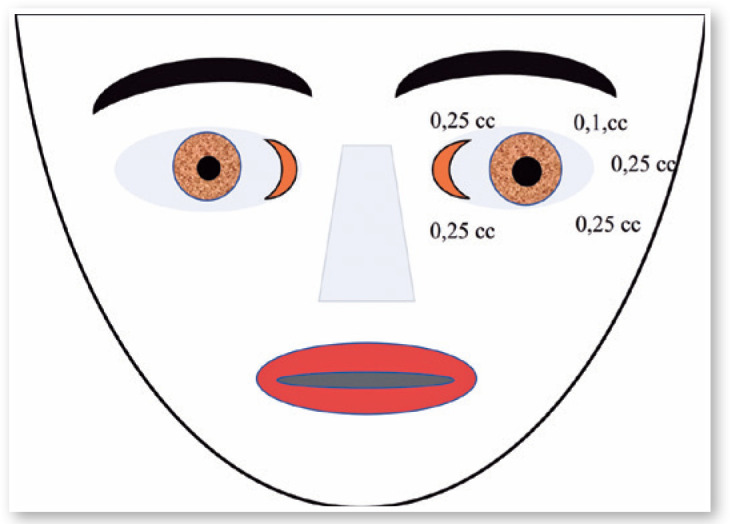
Drawing to show the places and doses of Botox injections for treating blepharospasm.

March 7, 1988: Oculinum was injected with good results (Figure 11). I repeated the injections 30 times, with intervals of 3 to 4 months, always with good results. I did not note a reduction of the effect. Only at the 20th injection did blepharoptosis occur, which disappeared after 15 days.

## CASE 5

**Figure 12:**
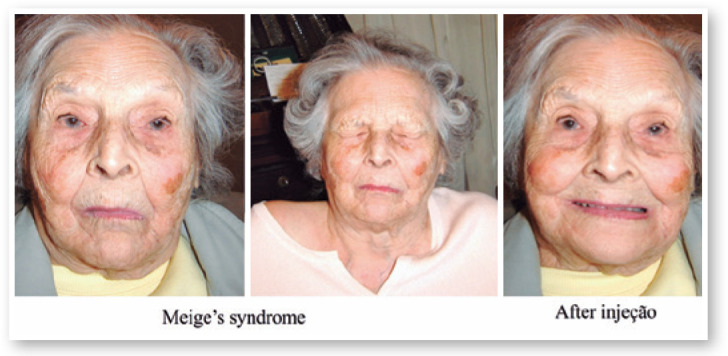
Patient with Meige´s syndrome before and after Botox injection.

October 28, 1992: M.A.C., female; closed eyes; jerking of the face, mouth, and neck; dysphonia (Figure 12).

The patient was taking clonazepam.

Diagnosis: Orofacial dystonia (Meige’s syndrome)

**Figure 13:**
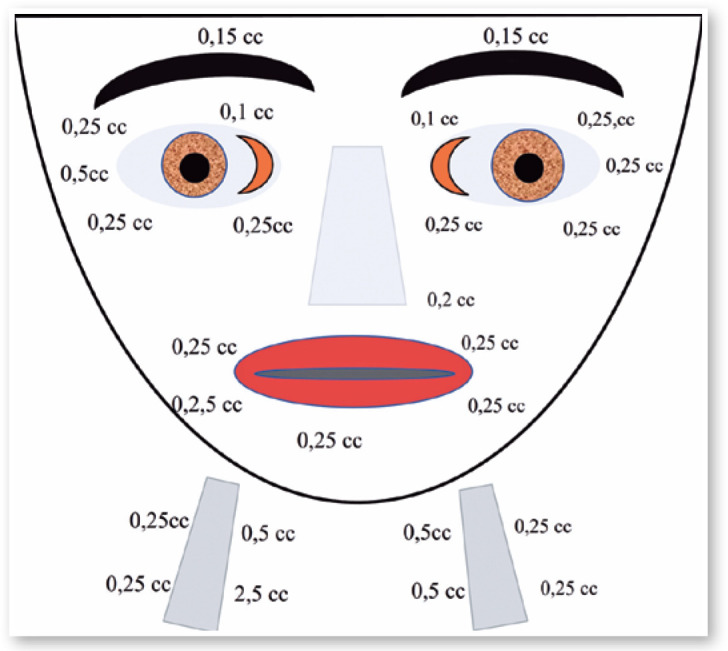
Doses and placement of Botox injections in the patient shown in Fgure 12.

Oculinum was injected bilaterally (Figure 13) in her face, mouth, and neck. The result was satisfactory; I repeated the injections 16 times with intervals of about 4 months. I did not observe a reduction in the effect.

## CASE 6

February 28, 1982: Over the past 16 years, the patient’s left eye had been closing. The patient could not see out of this eye.

**Figure 14:**
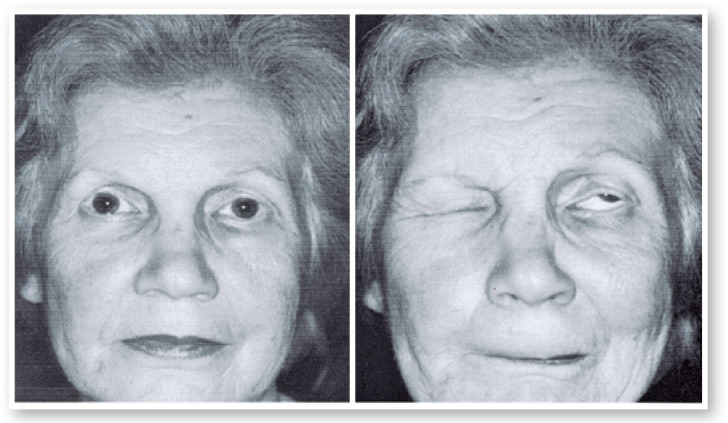
Patient with right blepharospasm. Right: interval between spasms.

Diagnosis: Left eye lid blepharospasm (Figure 14).

**Figure 15:**
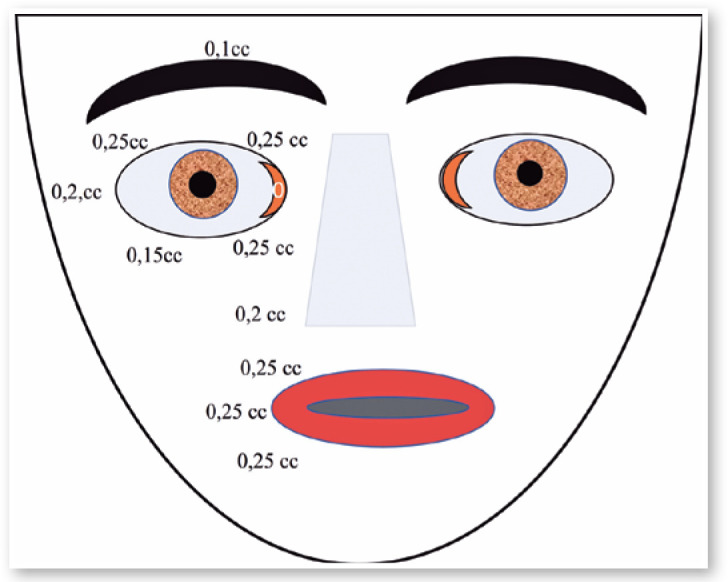
Places of injections and doses of Botox in the patient show in Figure 14.

Botox was injected around her left eye 21 times, with intervals of 3 to 4 months until 1999 (Figure 15). In 1998, I went to use Botox. I did not observe a reduction in the effect.

In the old days, treatment for strabismus in Brazil could be summarized as orthoptic, pleoptic, and surgery, until the day when I began to use botulinum toxin, which I brought to Brazil in 1981. Facial and palpebral dystonias were treated pharmacologically. Colleagues of the Escola Paulista de Medicina tried to use baclopheno^(4,5)^, but with meager results. Things have changed for physicians and their patients all around the world, thanks to the beautiful work of Alan B. Scott and Brazilian researchers with my initial work with Oculinum/Botox.
